# Codon Deviation Coefficient: a novel measure for estimating codon usage bias and its statistical significance

**DOI:** 10.1186/1471-2105-13-43

**Published:** 2012-03-22

**Authors:** Zhang Zhang, Jun Li, Peng Cui, Feng Ding, Ang Li, Jeffrey P Townsend, Jun Yu

**Affiliations:** 1 Computational Bioscience Research Center (CBRC), King Abdullah University of Science and Technology (KAUST), Thuwal 23955-6900, Kingdom of Saudi Arabia; 2 Current address: CAS Key Laboratory of Genome Sciences and Information, Beijing Institute of Genomics, Chinese Academy of Sciences, Beijing 100029, China; 3 School of Biological Sciences, The University of Hong Kong, Hong Kong, China; 4 Current address: Department of Pharmacology and Toxicology and the Cancer Center, Medical College of Wisconsin, Milwaukee, Wisconsin 53226, USA; 5 Department of Ecology and Evolutionary Biology, Yale University, New Haven, Connecticut 06520, USA; 6 Program in Computational Biology and Bioinformatics, Yale University, New Haven, Connecticut 06520, USA; 7 CAS Key Laboratory of Genome Sciences and Information, Beijing Institute of Genomics, Chinese Academy of Sciences, Beijing 100029, China

**Keywords:** Codon deviation coefficient, CDC, Codon usage bias, CUB, Statistical significance, Background nucleotide composition, GC content, Purine content, Bootstrapping

## Abstract

**Background:**

Genetic mutation, selective pressure for translational efficiency and accuracy, level of gene expression, and protein function through natural selection are all believed to lead to codon usage bias (CUB). Therefore, informative measurement of CUB is of fundamental importance to making inferences regarding gene function and genome evolution. However, extant measures of CUB have not fully accounted for the quantitative effect of background nucleotide composition and have not statistically evaluated the significance of CUB in sequence analysis.

**Results:**

Here we propose a novel measure--Codon Deviation Coefficient (CDC)--that provides an informative measurement of CUB and its statistical significance without requiring any prior knowledge. Unlike previous measures, CDC estimates CUB by accounting for background nucleotide compositions tailored to codon positions and adopts the bootstrapping to assess the statistical significance of CUB for any given sequence. We evaluate CDC by examining its effectiveness on simulated sequences and empirical data and show that CDC outperforms extant measures by achieving a more informative estimation of CUB and its statistical significance.

**Conclusions:**

As validated by both simulated and empirical data, CDC provides a highly informative quantification of CUB and its statistical significance, useful for determining comparative magnitudes and patterns of biased codon usage for genes or genomes with diverse sequence compositions.

## Background

Codon usage bias or CUB, a phenomenon in which synonymous codons (that encode the same amino acid) are used at different frequencies, is generally believed to be a combined outcome of mutation pressure, natural selection, and genetic drift [1-5]. Within any given species, genes often exhibit variable degrees of CUBs. Moreover, CUB for an individual gene is related closely with gene expression for translational efficiency and/or accuracy [6-10]. Therefore, the ability to accurately quantify CUBs for protein-coding sequences is of fundamental importance in revealing the underlying mechanisms behind codon usage and understanding gene evolution and function in general.

Over the past few years, a number of measures have been proposed for the quantification of CUB [11-23], leading to investigations on the pattern of CUBs within and across species [24-30]. Since CUB is primarily shaped by selection and mutation [5], different measures are differentially informative with regard to differentiating causes. For instance, there are purely descriptive measures of CUB as caused by the joint effects of mutation and selection, such as, the Effective Number of Codons (*N_c _*or ENC) [13] and the Relative Synonymous Codon Usage [22]. Alternatively, other measures of CUB specifically accord with selection on codon usage associated with translation, such as, the Codon Adaption Index (CAI) [12] and the Frequency of Optimal codons [15]. In addition, a number of studies have attempted to estimate selection on codon usage based on population genetics [31-35].

These existing measures generally fall into two categories, as they compare the observed codon usage distribution of target coding sequence against the distribution based on a reference set of highly-expressed genes (e.g., CAI) or the distribution based on a null hypothesis of uniform usage of different synonymous codons (e.g., *N_c_*). The former measures are highly dependent on their corresponding reference sets (from which preferred codons are derived) and accordingly are limited by the comprehensiveness and accuracy of reference sets. Since reference sets are species-specific, these measures are inappropriate for comparison of CUBs across species [36]. Additionally, they are unreliable in cases where there is inadequate knowledge about the highly-expressed genes for a given species [37], such as for newly sequenced species that have a limited number of annotated genes.

Due to these shortcomings, measures that do not require prior knowledge of reference gene sets have been implemented. These measures assume a null distribution of uniform usage of synonymous codons and estimate the departure of the observed codon usage from the expected. Among them, *N_c _*is one of the most widely used measures [13]. Its variant, *N_c_' *[19], incorporates GC content of coding sequence as background nucleotide composition (BNC) into CUB estimation. Accounting for BNC refines codon usage analysis, providing a comparable metric for analyses within and among species exhibiting various non-uniform BNCs. In the context of protein-coding sequences, for instance, bacteria have diverse BNCs as their GC contents vary widely - from ~20% to ~80%. Even within a single species, genes often differ considerably in background GC content, as in the case of *Escherichia coli str. K-12 substr. MG1655*, whose genes have GC contents ranging from 26.9% (*rfaS*; length = 311aa) to 66.8% (*yagF*; length = 655aa). Therefore, it is crucial to measure the departure of codon usage from the corresponding background composition (instead of the presumed uniform codon usage). Due to its appropriate consideration of BNC, *N_c_' *outperforms other relevant measures [19].

However, all extant measures (including *N_c_'*) still have limitations. First, they give a general estimate of CUB, but have not been supplied with straightforward procedures for assessing the statistical significance of the bias in codon usages for any given gene. Genes that vary in length and differ in CUB may exhibit different levels of statistical significance for their codon biases. Assessing statistical significance can strengthen functional relationships ascertained considerably by discounting sampling error in correlated gene sets. Second, no previous measure is fully effective at incorporating BNC into CUB estimation. Although *N_c_' *factors GC content as BNC, it does not account for known variation in BNCs at three different codon positions [38]. In bacteria, for instance, *Bartonella quintana str. Toulouse *and *Clostridium thermocellum ATCC 27405 *have very similar GC contents in coding sequences (40.5% and 40.4%, respectively), but their position-specific GC contents are quite different: 53.3% and 47.3% at the first codon position, 38.6% and 34.0% at the second codon position, and 29.5% and 39.9% at the third codon position, respectively. Likewise, genes within a given species can also have heterogeneous BNCs at the three codon positions; in *E. coli*, for example, there are two genes, *emrE *and *hlyE*, that are similar in their overall GC contents (41.5% and 41.1%) but different in positional GC contents: 42.7% and 48.2% at the first position, 46.4% and 32.0% at the second position, and 35.5% and 43.2% at the third position, respectively. Such differences in positional BNCs reflect the outcomes of diverse evolutionary mechanisms (e.g., dinucleotide bias [39], horizontal gene transfer [40], strand compositional asymmetry in bacteria [41], isochore structure in vertebrates [42], etc.), thus conflating the roles of mutation and selection acting at different codon positions. Therefore, incorporation of differential positional BNCs into CUB estimation promises to increase its effectiveness and reliability.

Moreover, GC content is not the sole parameter of BNC. As illustrated in Zhang and Yu [43], joint use of GC and purine contents effectively models nucleotide, codon, and amino acid compositions. In contrast to a broader variation of GC content, purine content varies within a much narrower range fluctuating around 50%, presumably because purines play a determinative role in physicochemical properties of amino acids [44,45]. Similar with GC content, purine content differs not only from one species to another, but also from one gene to another, and even between genes with similar GC contents. For instance, *emrE *and *hlyE *in *E. coli*, which are similar in their overall GC contents, have entirely different purine contents not only at the overall level (45.8% and 55.6%, respectively), but also at three codon positions (54.5% and 68.3% at the first position, 34.5% and 48.2% at the second position, and 48.2% and 50.2% at the third position, respectively). Thus, in addition to GC content, purine content is also a significant feature of BNC.

Here we present a novel measure, Codon Deviation Coefficient (CDC), using it to characterize CUB and to ascertain its statistical significance. CDC takes account of both GC and purine contents, comprehensively addressing heterogeneous BNCs, not only in sequences but also at three codon positions. It adopts the cosine distance metric to quantify CUB and employs the bootstrapping to assess its statistical significance, requiring no prior knowledge of reference gene sets. We describe CDC in detail and provide comparative results in the form of an in-depth evaluation of simulated sequences and empirical data.

## Methods

### Expected codon usage

CDC considers both GC and purine contents as BNC and derives expected codon usage from observed positional GC and purine contents. We denote the content of the four nucleotides (adenine, thymine, guanine, and cytosine), GC content, and purine content as *A*, *T*, *G*, *C*, *S *and *R*, respectively. As in Zhang and Yu [43], position-dependent nucleotide contents can be formulated in the following way:

(1) $$A_{i}=\left(1-S_{i}\right)R_{i},T_{i}=\left(1-S_{i}\text{)(}1-R_{i}\right),G_{i}=S_{i}R_{i},C_{i}=S_{i}\left(1-R_{i}\right),$$

where *S_i _*and *R_i _*are their corresponding observed contents at codon position *i *and *A_i_*, *T_i_*, *G_i_*, *C_i _*are expected nucleotide contents at codon position *i *(*i *= 1, 2, 3). For any sense codon *xyz*, where *x*, *y*, *z *∈ {A, T, G, C}, the expected usage *πxyz *is defined as the product of its constituent expected nucleotide contents *x*_1_*y*_2_*z*_3_, normalized by the sum over all sense codons, viz.

(2) $$\pi_{xyz}=\frac{x_{1}y_{2}z_{3}}{\underset{abc}{\sum }w_{abc}a_{1}b_{2}c_{3}},$$

$$\text{where }w_{abc}=\left\{\begin{array}{c} 1\text{,if }abc\text{ is a sense codon}\\ 0,\text{ otherwise} \end{array}\right)\text{ and }a,b,c\in \left\{\text{A},\text{T},\text{G},\text{C}\right\}.$$

### Codon usage bias

Any coding sequence can be represented as a vector of *n *dimensions, whose entries correspond to *n *sense codon usages in the sequence. The dimension *n *equals 61 for the canonical code; although codons ATG and TGG could be set aside due to the absence of synonymous codons, calculation based on a vector of 61 dimensions instead of 59 dimensions makes little substantial difference. To calculate CUB for any given sequence, we employ the cosine distance metric [46] based on the cosine of the angle between the two vectors of *n *dimensions. Therefore, when both expected (π) and observed ($\hat{\pi }$) codon usage vectors are available for any given sequence, CDC renders a distance coefficient ranging from 0 (no bias) to 1 (maximum bias), to represent CUB, expressed by the deviation of $\hat{\pi }$ from π (Eq. 3).

(3) $$\text{CDC}=1-\frac{\sum_{xyz}\pi_{xyz}\times {\hat{\pi }}_{xyz}}{\sqrt{\sum_{xyz}\pi_{xyz}{}^{2}\times \sum_{xyz}{\hat{\pi }}_{xyz}{}^{2}}}$$

### Statistical significance of codon usage bias

We implement a bootstrap resampling of *N *= 10000 replicates for any given sequence to evaluate the statistical significance of non-uniform codon usage. Each replicate is randomly generated according to the sequence BNC (*S_i _*and *R_i_*, *i *= 1, 2, 3) and the sequence length. Consequently, we obtain a bootstrap distribution of *N *estimates of CUB. A two-sided bootstrap *P*-value is calculated as twice the smaller of the two one-sided *P*-values [47]. *P *ranges from 0 to 1. By convention, a statistically significant CUB is identified by *P *< 0.05. CDC features its first application of the bootstrap resampling in estimating the statistical significance of CUB. Bootstrapping may also be applicable to other related measures.

### Implementation and availability

CDC is written in standard C++ programming language and implemented into Composition Analysis Toolkit (CAT), which is distributed as open-source software and licensed under the GNU General Public License. Its software package, including compiled executables on Linux/Mac/Windows, example data, documentation, and source codes, is freely available at http://cbb.big.ac.cn/software and http://cbrc.kaust.edu.sa/CAT.

## Results and discussion

### Comparative analysis on simulated data

To evaluate the performance of CDC and compare it against the most powerful extant measure, *N_c_'*, as well as *N_c_*, we took an approach based on that of Novembre [19] to simulate coding sequences specifying different positional BNCs and varying sequence lengths. Five sets of position-associated compositions were used to generate simulated sequences (Table 1). It should be noted that CDC ranges from 0 (no bias) to 1 (maximum bias), whereas *N_c_' *and *N_c _*range from 20 (maximum bias) to 61 (no bias). To facilitate comparisons of CDC with *N_c_' *and *N_c_*, we use the formula (61- *N_c_'*)/41 and (61- *N_c_*)/41 to rescale their ranges, denoted as scaled *N_c_' *and scaled *N_c_*, respectively, from 0 (no bias) to 1 (maximum bias).

**Table 1:** Background nucleotide compositions at three codon positions specified in simulations

Content	None	Low	Med-1	Med-2	High
1st position	0.5	0.5	0.5	0.5	0.5

2nd position	0.5	0.4	0.3	0.2	0.1

3rd position	0.5	0.6	0.7	0.8	0.9

**Figure 1:**
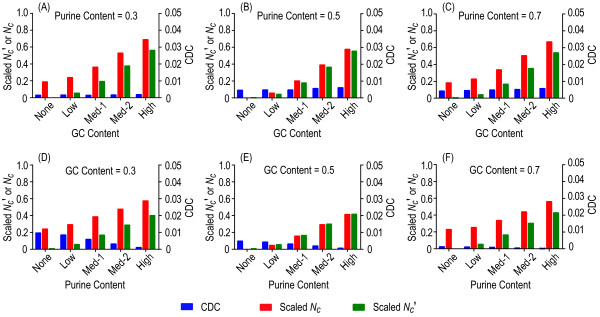
**Codon usage bias across a variety of positional background nucleotide compositions**. Heterogeneous positional background compositions were considered for GC content (panels A to C) and purine content (panels D to E), respectively. The expected values of codon usage bias are zero for all examined cases.

A good measure should not deviate much from its expectation as the amount of data approaches infinity or any sufficiently large number. Thus, we first simulated sequences with a total of 100,000 codons using five positional composition sets (PCSs) (Table 1). Considering the fact that both GC and purine contents govern BNC, we fixed one of them to be uniform at three codon positions and allowed the other to have various positional compositions. We examined heterogeneous positional compositions for GC (Figure 1A to 1C) and purine (Figure 1D to 1F) contents, respectively. Consistent with expectations, when the PCS was uniform, CDC and scaled *N_c_' *performed similarly, both taking a value close to 0 (Figure 1). When the heterogeneity of positional composition increased for GC content (Figure 1A to 1C), CDC continued to perform well for all cases examined, whereas scaled *N_c_' *and scaled *N_c _*generated biased estimates, especially in cases where there was high heterogeneity in positional BNCs. Similarly, when purine content had heterogeneous positional compositions (Figure 1D to 1F), CDC again exhibited much lower biases than scaled *N_c_' *and scaled *N_c_*. Since *N_c _*ignores BNC, *N_c_' *performed better than *N_c _*when the PCS was non-uniform (Figure 1A, C, D and 1F) and they exhibited comparable estimates only in cases where the PCS was uniform (Figure 1B and 1E). These results agree well with those of Novembre [19]. In addition, when we set heterogeneous positional BNCs for both GC and purine contents, CDC consistently outperformed *N_c_' *and *N_c _*for nearly all the parameter combinations tested (Table 2).

**Table 2:** Codon usage bias across a variety of positional background compositions for GC and purine contents

GC Content	Purine Content	CDC	Scaled *N_c_*	Scaled *N_c_'*
None	None	0.00452	0.00001	0.00186
	
	Low	0.00407	0.04843	0.05557
	
	Med-1	0.00302	0.15130	0.15968
	
	Med-2	0.00164	0.28613	0.29389
	
	High	0.00054	0.40797	0.41146

Low	None	0.00452	0.05505	0.04181
	
	Low	0.00411	0.09548	0.08752
	
	Med-1	0.00305	0.19808	0.19091
	
	Med-2	0.00164	0.31892	0.31461
	
	High	0.00060	0.44778	0.44199

Med-1	None	0.00486	0.20367	0.17790
	
	Low	0.00438	0.23485	0.21262
	
	Med-1	0.00305	0.31876	0.29478
	
	Med-2	0.00203	0.42851	0.40322
	
	High	0.00054	0.53585	0.51978

Med-2	None	0.00529	0.38525	0.36068
	
	Low	0.00460	0.40628	0.38358
	
	Med-1	0.00337	0.47542	0.43927
	
	Med-2	0.00182	0.56759	0.52569
	
	High	0.00056	0.65842	0.62645

High	None	0.00606	0.56671	0.54706
	
	Low	0.00520	0.59091	0.56666
	
	Med-1	0.00371	0.65926	0.61789
	
	Med-2	0.00225	0.71856	0.66928
	
	High	0.00065	0.77246	0.73600

Sequences with 100000 codons were simulated. The expected value of codon usage bias is zero so that these estimated values are also the deviations from the expected.

To evaluate CDC in a comprehensive manner, we also examined all possible quantitative relationships among positional GC contents (Table 3), although there are identified patterns about quantitative relationships among positional nucleotide compositions (e.g., GC content at the 1st codon position tends to be always larger than that at the 2nd codon position [48]). On the whole, CDC achieved greater power than scaled *N_c_' *and scaled *N_c _*across all examined cases. Scaled *N_c_' *performed better than scaled *N_c_*, consisting again with the analysis reported by Novembre [19]. Similar results were also obtained when we considered all possible quantitative relationships among positional purine contents (Table 4).

**Table 3:** Codon usage bias across all possible quantitative relationships among positional GC contents

GC content			Purine content = 0.3			Purine content = 0.5			Purine content = 0.7		
**1st**	**2nd**	**3rd**	**CDC**	**Scaled** ***N_c_***	**Scaled** ***N_c_*'**	**CDC**	**Scaled** ***N_c_***	**Scaled** ***N_c_*'**	**CDC**	**Scaled** ***N_c_***	**Scaled** ***N_c_*'**

0.3	0.5	0.7	0.00153	0.34160	0.23472	0.00586	0.24586	0.23332	0.00481	0.39716	0.21314

0.3	0.7	0.5	0.00147	0.15648	0.05716	0.00551	0.04827	0.06330	0.00498	0.24616	0.05866

0.5	0.3	0.7	0.00146	0.36662	0.19363	0.00470	0.20034	0.17544	0.00441	0.34555	0.17306

0.5	0.7	0.3	0.00143	0.35276	0.21224	0.00519	0.19619	0.21974	0.00417	0.34831	0.21815

0.7	0.3	0.5	0.00069	0.21330	0.01419	0.00236	0.02999	0.02692	0.00233	0.16172	0.03574

0.7	0.5	0.3	0.00066	0.38224	0.22121	0.00257	0.22392	0.23947	0.00236	0.33561	0.24588

Sequences with 100000 codons were simulated. The compositions in the Med-1 set (0.3, 0.5 and 0.7) were used. GC content was considered non-uniform at three codon positions, whereas purine content was set uniform at three codon positions. The expected value of codon usage bias is zero so that these estimated values are also the deviations from the expected.

**Table 4:** Codon usage bias across all possible quantitative relationships among positional purine contents

Purine content			GC content = 0.3			GC content = 0.5			GC content = 0.7		
**1st**	**2nd**	**3rd**	**CDC**	**Scaled** ***N_c_***	**Scaled** ***N_c_*'**	**CDC**	**Scaled** ***N_c_***	**Scaled** ***N_c_*'**	**CDC**	**Scaled** ***N_c_***	**Scaled** ***N_c_*'**

0.3	0.5	0.7	0.01743	0.35780	0.18606	0.01023	0.15974	0.17789	0.00232	0.34949	0.17267

0.3	0.7	0.5	0.01836	0.21922	0.01880	0.01036	0.01515	0.01520	0.00263	0.24157	0.00941

0.5	0.3	0.7	0.00616	0.38200	0.16209	0.00294	0.15248	0.16112	0.00063	0.33321	0.16601

0.5	0.7	0.3	0.00566	0.31973	0.15002	0.00302	0.16556	0.15842	0.00061	0.37234	0.15754

0.7	0.3	0.5	0.00182	0.27781	0.02340	0.00079	0.02564	0.02805	0.00026	0.21360	0.02756

0.7	0.5	0.3	0.00179	0.35410	0.15793	0.00087	0.16099	0.15939	0.00024	0.35439	0.15404

Sequences with 100000 codons were simulated. The compositions in the Med-1 set (0.3, 0.5 and 0.7) were used. Purine content was considered non-uniform at three codon positions, whereas GC content was set uniform at three codon positions. The expected value of codon usage bias is zero so that these estimated values are also the deviations from the expected.

**Figure 2:**
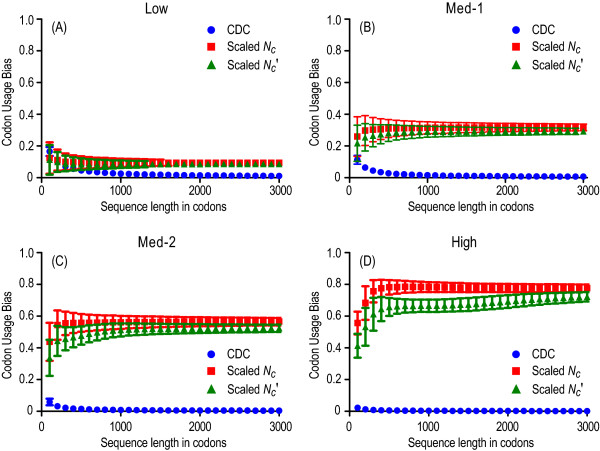
**Codon usage bias across a range of sequence lengths**. Sequences were simulated with the four non-uniform positional composition sets: Low (panel A), Med-1 (panel B), Med-2 (panel C) and High (panel D). Each estimate was determined based on 10000 replicate simulated sequences. The expected values of codon usage bias are zero for all examined cases.

To examine the effect of variable sequence length on the integrity of CDC, we considered a wide range of sequence lengths from 100 to 3,000 codons. We set both GC and purine contents to be heterogeneous at three codon position using the four non-uniform PCSs (Table 1). To avoid stochastic errors, we repeated simulations 10,000 times for each parameter combination and thus each estimate was determined from 10,000 replicates. Overall, CDC performed better than *N_c_*' and *N_c _*across all sequence lengths examined (Figure 2). When the heterogeneity of BNC increased from low to high, CDC tended to have less biases, whereas *N_c_*' and *N_c _*produced increasingly biased estimates, especially for the case where there was high heterogeneity in positional BNCs (Figure 2D). For short sequences (<300 codons), CDC yielded much lower biases and smaller standard deviations (SD) than *N_c_' *and *N_c_*, although all three measures produced estimates that are somewhat biased. To obtain more reliable estimates of CUB, our results suggest that input sequences should have at least 100 codons in length. When sequence length was decreased below 100 codons, CDC still performed better than *N_c_' *and *N_c_*, although the biases of *N_c_' *and *N_c _*were in opposite directions as compared with those of CDC (Figure 2B to 2D; not apparent in Figure 2A). For long sequences, CDC generated less biased estimates and SDs, whereas *N_c_' *and *N_c _*continued to yield more biased estimates and SDs.

To test the influence of different CUBs on the power of CDC, we evaluated a range of CUBs from low to high. Unlike the previous simulations (which are based on nucleotide compositions), we generated simulated sequences by randomly setting different synonymous codon frequencies and considering variable CUBs with a range from 0.1 to 0.9. We repeated simulations 1,000 times for each case and accordingly each estimate was averaged over 1,000 replicates. On the whole, CDC exhibited greater power in detecting diverse CUBs; compared with *N_c_*' and *N_c_*, the estimated CUBs of CDC were very closer to the expected ones (Table 5). When the expected CUBs varied from low to high, CDC performed consistently to give rise to close estimates. Contrastingly, *N_c_*' and *N_c _*yielded biased CUB estimates across all tested cases and these biases became more pronounced when the expected CUB was extremely low. When the expected CUBs increased from low to high, *N_c_*' and *N_c _*exhibited increasing power in CUB estimation. While they approached the power of CDC when the expected CUB was high, CDC remained more powerful than *N_c_*' and *N_c_*. Taken together, our simulation results demonstrated that CDC is superior to *N_c_' *and *N_c_*.

**Table 5:** Differences between estimated and expected codon usage biases

Expected CUB	(Estimated CUB)*^a ^*- (Expected CUB)		
	
	CDC	Scaled *N_c_*	Scaled *N_c_*'
0.1	0.00137	0.60854	0.61438

0.2	0.00174	0.47951	0.52490

0.3	-0.00245	0.38428	0.43524

0.4	0.00186	0.27647	0.35793

0.5	-0.00060	0.17750	0.21300

0.6	0.00437	0.08031	0.15215

0.7	0.00542	0.01312	0.06657

0.8	-0.00014	0.04816	-0.02663

0.9*^b^*	-	-	-

*^a^*Each estimate was averaged over 1000 replicate simulated sequences that each had 100000 codons.

*^b^*Sequences with the expected codon usage bias at 0.9 were not possible to successfully simulate.

### Application to empirical data

It is generally acknowledged that CUB correlates closely with gene expression level in both unicellular [6-10] and multicellular [11,49-51] organisms. Different species may have different heterogeneities in positional BNCs. To empirically test CDC and compare it to three popular measures, *N_c_'*, *N_c _*and CAI, we collected multiple expression data sets from five different species in this study: (1) *Escherichia coli *from Bernstein et al. [52] (in LB and M9 media), (2) *Saccharomyces cerevisiae *from Holstege et al. [53], (3) *Drosophila melanogaster *from Zhang et al. [54], (4) *Caenorhabditis elegans *from Roy et al. [55], and (5) *Arabidopsis thaliana *from Wuest et al. [56] (Additional file 1). We estimated CUB by CDC, scaled *N_c_*', scaled *N_c _*and CAI, and correlated their estimates with gene expression levels in these five species (Table 6).

**Table 6:** Correlation coefficients of codon usage bias with gene expression level

Data*^a^*	*E. coli*^1^		*S. cerevisiae*^2^	*D. melanogaster^3^*	*C. elegans*^4^	*A. thaliana*^5^
	
	LB (*n *= 1762*^b^*)	M9 (*n *= 2766*^b^*)	(*n *= 5142*^b^*)	(*n *= 1651*^b^*)	(*n *= 12184*^b^*)	(*n *= 1332*^b^*)
CDC*^c^*	0.433	0.367	0.654	0.460	0.374	0.228

Scaled *N_c_'^c^*	0.315	0.187	0.664	0.302	0.328	0.130

Scaled *N_c_^c^*	0.257	0.125	0.600	0.321	0.192	0.063

CAI*^c^*	0.443	0.288	0.675	0.386	-0.118	0.034

*^a^*Expression data were obtained from ^1^Bernstein et al., ^2^Holstege et al., ^3^Zhang et al., ^4^Roy et al., and ^5^Wuest et al. (see details in Additional file 1).

*^b^*Number of genes (*n*).

*^c^P *< 0.0001 for all values.

On the whole, CDC outperformed scaled *N_c_*' and scaled *N_c _*in correlating closely with gene expression level. Although CDC and scaled *N_c_*' produced comparable correlation coefficients in yeast (detailed below), CDC exhibited larger correlation coefficients than scaled *N_c_*' and scaled *N_c _*for all the rest cases (Table 6). When comparing CDC to CAI, we found comparable correlation coefficients in *E. coli *(LB medium) and yeast, but in general CDC performed better than CAI (Table 6 and Additional file 1). However, it should be noticed that the values of CAI are calculated from expression data (since it requires a reference set of highly-expressed genes), whereas those of CDC are not. When we restricted the above analysis to the top 10% genes referring to their expression levels, CDC continued to perform better than scaled *N_c_*', scaled *N_c_*, and CAI (Additional file 1). In addition, considering the correlation coefficients among these five species, we found that the smallest values always belonged to *A. thaliana *(regardless of metric used), indicating relatively weaker selection on *A. thaliana *codon usage by comparison with those of the other four species (Table 6). Such phenomenon was discovered previously in a comparative analysis between *A. thaliana *and *Oryza sativa *[57]. Overall, CDC correlated positively with gene expression level, much better than scaled *N_c_*', scaled *N_c_*, and CAI.

**Figure 3:**
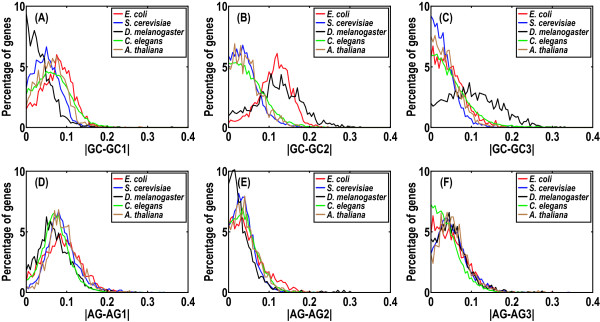
**Heterogeneity of positional background nucleotide compositions in *E. coli *(2,766 genes in M9 medium), *S. cerevisiae *(5,142 genes), *D. melanogaster *(1,651 genes),*C. elegans *(12,184 genes), and *A. thaliana *(1,332 genes)**. Heterogeneities of positional GC contents are represented by absolute differences between overall GC content and its positional contents: GC-GC1 for the first position (panel A), GC-GC2 for the second position (panel B), and GC-GC3 for the third position (panel C), respectively. Likewise, heterogeneities of positional purine content are absolute differences between overall purine (AG) content and its positional contents: AG-AG1 for the first position (panel D), AG-AG2 for the second position (panel E), and AG-AG3 for the third position (panel F), respectively.

As noted, the correlation coefficients produced by CDC and scaled *N_c_*' were similar in yeast but different in others (Table 6). Since CDC takes positional GC and purine contents as BNC and *N_c_*' considers only GC content as BNC and ignores positional heterogeneity, this result can be probably explained by relatively lower heterogeneity of positional BNCs in yeast. To further investigate this possibility, we examined the heterogeneities of positional GC and purine contents in these five species (Figure 3). Consistent with our expectation, heterogeneities of positional GC contents were indeed lower in yeast by comparison with other species (Figure 3A to 3C), especially at the second and third codon positions. In contrast, higher heterogeneities of positional GC contents were apparent in *E. coli *(Figure 3A and 3B for the first and second codon positions, respectively) and *D. melanogaster *(Figure 3B and 3C for the second and third codon positions, respectively). These results agree well with the observation that the difference of correlation coefficient between CDC and scaled *N_c_*' in yeast was smaller than that in *E. coli *or *D. melanogaster *(Table 6). As a consequence, CDC correlated more closely with scaled *N_c_*' in yeast than in *E. coli *or *D. melanogaster *(Figure S13 in Additional file 1). In contrast to GC content, heterogeneities of positional purine contents were relatively smaller and similar among the five species tested, presumably attributable to the fact that GC content ranges more broadly (20%--80%) than purine content (40%--60%) [48,58,59].

**Figure 4:**
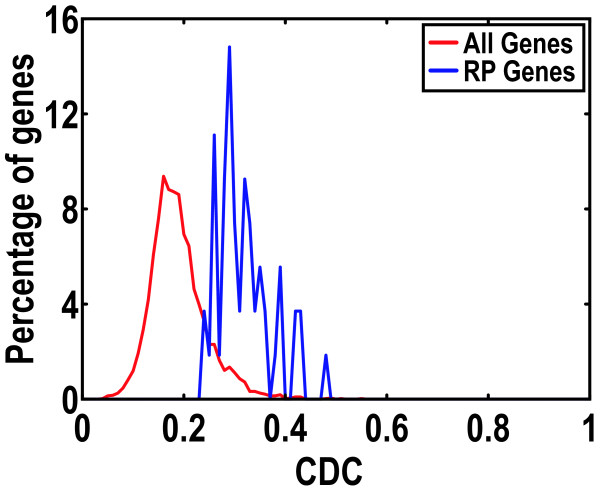
**Comparison of CDC distributions between ribosomal protein (54 RP genes vary from 0.244 to 0.481) genes and all genes (4,144 genes range from 0.046 to 0.550) in *E. coli***.

We proceeded to calculate CDC values (as well as GC and purine contents) for all *E. coli *genes (Additional file 2). CDC values ranged from 0.046 to 0.550 and the mean and median values were 0.239 and 0.187, respectively (Figure 4). The majority of genes (69%) exhibited CDC values between 0.15 and 0.25. The gene with the highest CDC value is *trpL*, a key component in the attenuation system that controls the expression of the trpLEDCBA operon in response to tryptophan availability [60]. However, bootstrap resampling illustrates that the CUB value of *trpL *gene is not statistically significant (*P *= 0.77), most likely due to its short length (14 aa), consistent with our simulation results that short sequences tend to have biased CUB estimates. The gene with the highest CDC value and statistical significance in CUB is *rpmI *(CDC = 0.481), which encodes ribosomal protein L35. In contrast, scaled *N_c_' *and scaled *N_c _*identified *rplL *(encoding the ribosomal protein L7/L12) and *eno *(catalyzing the interconversion of 2-phosphoglycerate and phosphoenolpyruvate) genes, respectively, as having the strongest CUBs (Additional file 2).

Ribosomal protein (RP) genes are, in general, both essential and highly expressed, and it is believed that their CUB values are greater than those of other genes [61]. In the case of *E. coli*, CDC values for 54 RP genes vary from 0.244 to 0.481, larger than the mean and median values of all *E. coli *genes (Figure 4). Nearly all RP genes have statistically significant CUBs, with three exceptions (Additional file 3): (1) *rpmE*: CDC = 0.267, *P *= 0.1136; encoding RP L31, which may be loosely associated with ribosome [62], (2) *rpmF*: CDC = 0.329, *P *= 0.1096; encoding RP L32, which locates near the peptidyltransferase center [63], and (3) *rpmJ: *CDC = 0.422, *P *= 0.0564; encoding RP L36, which is non-essential for protein synthesis [64]. These results suggest that an accurate measure such as CDC has the potential to illuminate the evolutionary process that has operated on each gene.

## Conclusions

In summary, we have described a novel measure of CUB, the Codon Deviation Coefficient. As validated by simulated sequences and empirical data, CDC outperforms other measures by providing informative estimates of CUB and its statistical significance. CDC features no necessity for any prior knowledge regarding gene expression or function, properly accounts for BNC, and utilizes a bootstrap assessment to evaluate the statistical significance of CUB. Therefore, CDC promises a significant advance in raw analysis of codon usage, providing the means to better reveal aspects of the historical evolutionary pressures on gene function without the assumptions of underlying reference data sets.

## Abbreviations

CUB: Codon Usage Bias; CDC: Codon Deviation Coefficient; BNC: Background Nucleotide Composition; PCS: Positional Composition Set; *A*: Adenine content; *T*: Thymine content; *G*: Guanine content; *C*: Cytosine content; *S*: GC content; *R*: Purine content; *A_i_*, *T_i_*, *G_i_*, *C_i_*, *S_i_*, *R_i_*, *A*, *T*, *G*, *C*, *S*, *R *at codon position *i*, respectively, where *i *= 1, 2, 3.

## Competing interests

The authors declare that they have no competing interests.

## Authors' contributions

ZZ designed the algorithm, developed the program, and drafted the manuscript. JL participated in the design of the algorithm and analyzed the simulated data. PC collected expression data and analyzed the real data. FD carried out data visualization. AL participated in the software development and testing. JPT helped to draft the manuscript and revised the manuscript. JY supervised the study and revised the manuscript. All authors read and approved the final manuscript.

## Supplementary Material

Additional file 1 **Empirical expression data analysis**. Correlations between codon usage bias and gene expression level in different expression data sets. Click here for file

Additional file 2 **Estimates of codon usage bias for all E. coli genes**. Codon usage biases of all E. coli genes estimated by CDC, *N_c_*' and *N_c_*. Click here for file

Additional file 3 **Estimates of codon usage bias for ribosomal proteins in E. coli**. Codon usage biases of ribosomal proteins in E. coli estimated by CDC, *N_c_*' and *N_c_*. Click here for file

## References

[B1] Bulmer M The selection-mutation-drift theory of synonymous codon usage Genetics 1991 129 3 897 907 1752426 10.1093/genetics/129.3.897 PMC1204756

[B2] Akashi H Codon bias evolution in Drosophila. Population genetics of mutation-selection drift Gene 1997 205 1-2 269 278 10.1016/S0378-1119(97)00400-9 9461401

[B3] Chen SL Lee W Hottes AK Shapiro L McAdams HH Codon usage between genomes is constrained by genome-wide mutational processes Proc Natl Acad Sci USA 2004 101 10 3480 3485 10.1073/pnas.0307827100 14990797 PMC373487

[B4] Hershberg R Petrov DA Selection on codon bias Annu Rev Genet 2008 42 287 299 10.1146/annurev.genet.42.110807.091442 18983258

[B5] Plotkin JB Kudla G Synonymous but not the same: the causes and consequences of codon bias Nat Rev Genet 2011 12 1 32 42 10.1038/nrg2899 21102527 PMC3074964

[B6] Gouy M Gautier C Codon usage in bacteria: correlation with gene expressivity Nucleic Acids Res 1982 10 22 7055 7074 10.1093/nar/10.22.7055 6760125 PMC326988

[B7] dos Reis M Wernisch L Savva R Unexpected correlations between gene expression and codon usage bias from microarray data for the whole Escherichia coli K-12 genome Nucleic Acids Res 2003 31 23 6976 6985 10.1093/nar/gkg897 14627830 PMC290265

[B8] Goetz RM Fuglsang A Correlation of codon bias measures with mRNA levels: analysis of transcriptome data from Escherichia coli Biochem Biophys Res Commun 2005 327 1 4 7 10.1016/j.bbrc.2004.11.134 15629421

[B9] Coghlan A Wolfe KH Relationship of codon bias to mRNA concentration and protein length in Saccharomyces cerevisiae Yeast 2000 16 12 1131 1145 10.1002/1097-0061(20000915)16:12<1131::AID-YEA609>3.0.CO;2-F 10953085

[B10] Ghaemmaghami S Huh WK Bower K Howson RW Belle A Dephoure N O'Shea EK Weissman JS Global analysis of protein expression in yeast Nature 2003 425 6959 737 741 10.1038/nature02046 14562106

[B11] Shields DC Sharp PM Higgins DG Wright F "Silent" sites in Drosophila genes are not neutral: evidence of selection among synonymous codons Mol Biol Evol 1988 5 6 704 716 3146682 10.1093/oxfordjournals.molbev.a040525

[B12] Sharp PM Li WH The codon Adaptation Index-a measure of directional synonymous codon usage bias, and its potential applications Nucleic Acids Res 1987 15 3 1281 1295 10.1093/nar/15.3.1281 3547335 PMC340524

[B13] Wright F The 'effective number of codons' used in a gene Gene 1990 87 1 23 29 10.1016/0378-1119(90)90491-9 2110097

[B14] Morton BR Chloroplast DNA Codon Use - Evidence for Selection at the Psb-a Locus Based on Transfer-Rna Availability Journal of Molecular Evolution 1993 37 3 273 280 8230251 10.1007/BF00175504

[B15] Ikemura T Correlation between the abundance of Escherichia coli transfer RNAs and the occurrence of the respective codons in its protein genes: a proposal for a synonymous codon choice that is optimal for the *E. coli *translational system J Mol Biol 1981 151 3 389 409 10.1016/0022-2836(81)90003-6 6175758

[B16] Xia X An improved implementation of codon adaptation index Evol Bioinform Online 2007 3 53 58 19461982 PMC2684136

[B17] Suzuki H Brown CJ Forney LJ Top EM Comparison of correspondence analysis methods for synonymous codon usage in bacteria DNA Res 2008 15 6 357 365 10.1093/dnares/dsn028 18940873 PMC2608848

[B18] Supek F Vlahovicek K Comparison of codon usage measures and their applicability in prediction of microbial gene expressivity BMC Bioinformatics 2005 6 182 10.1186/1471-2105-6-182 16029499 PMC1199580

[B19] Novembre JA Accounting for background nucleotide composition when measuring codon usage bias Mol Biol Evol 2002 19 8 1390 1394 10.1093/oxfordjournals.molbev.a004201 12140252

[B20] Zeeberg B Shannon information theoretic computation of synonymous codon usage biases in coding regions of human and mouse genomes Genome Res 2002 12 6 944 955 10.1101/gr.213402 12045147 PMC1383734

[B21] Urrutia AO Hurst LD Codon usage bias covaries with expression breadth and the rate of synonymous evolution in humans, but this is not evidence for selection Genetics 2001 159 3 1191 1199 11729162 10.1093/genetics/159.3.1191 PMC1461876

[B22] Sharp PM Tuohy TM Mosurski KR Codon usage in yeast: cluster analysis clearly differentiates highly and lowly expressed genes Nucleic Acids Res 1986 14 13 5125 5143 10.1093/nar/14.13.5125 3526280 PMC311530

[B23] Angellotti MC Bhuiyan SB Chen G Wan XF CodonO: codon usage bias analysis within and across genomes Nucleic Acids Res 2007 35 Web Server W132 136 17537810 10.1093/nar/gkm392 PMC1933134

[B24] Cutter AD Wasmuth JD Blaxter ML The evolution of biased codon and amino acid usage in nematode genomes Mol Biol Evol 2006 23 12 2303 2315 10.1093/molbev/msl097 16936139

[B25] Cutter AD Wasmuth JD Washington NL Patterns of molecular evolution in Caenorhabditis preclude ancient origins of selfing Genetics 2008 178 4 2093 2104 10.1534/genetics.107.085787 18430935 PMC2323799

[B26] Herbeck JT Novembre J Codon usage patterns in cytochrome oxidase I across multiple insect orders J Mol Evol 2003 56 6 691 701 10.1007/s00239-002-2437-7 12911032

[B27] Ingvarsson PK Molecular evolution of synonymous codon usage in Populus BMC Evol Biol 2008 8 307 10.1186/1471-2148-8-307 18983655 PMC2586637

[B28] Powell JR Moriyama EN Evolution of codon usage bias in Drosophila Proc Natl Acad Sci USA 1997 94 15 7784 7790 10.1073/pnas.94.15.7784 9223264 PMC33704

[B29] Qiu S Bergero R Zeng K Charlesworth D Patterns of codon usage bias in Silene latifolia Mol Biol Evol 2011 28 1 771 780 10.1093/molbev/msq251 20855431

[B30] Vicario S Moriyama EN Powell JR Codon usage in twelve species of Drosophila BMC Evol Biol 2007 7 226 10.1186/1471-2148-7-226 18005411 PMC2213667

[B31] Akashi H Inferring weak selection from patterns of polymorphism and divergence at "silent" sites in Drosophila DNA Genetics 1995 139 2 1067 1076 7713409 10.1093/genetics/139.2.1067 PMC1206357

[B32] Sharp PM Bailes E Grocock RJ Peden JF Sockett RE Variation in the strength of selected codon usage bias among bacteria Nucleic Acids Res 2005 33 4 1141 1153 10.1093/nar/gki242 15728743 PMC549432

[B33] dos Reis M Wernisch L Estimating translational selection in eukaryotic genomes Mol Biol Evol 2009 26 2 451 461 10.1093/molbev/msn272 19033257 PMC2639113

[B34] Zeng K Charlesworth B Estimating selection intensity on synonymous codon usage in a nonequilibrium population Genetics 2009 183 2 651 662 10.1534/genetics.109.101782 19620398 PMC2766324

[B35] Haddrill PR Zeng K Charlesworth B Determinants of synonymous and nonsynonymous variability in three species of Drosophila Mol Biol Evol 2011 28 5 1731 1743 10.1093/molbev/msq354 21191087

[B36] Ermolaeva MD Synonymous codon usage in bacteria Curr Issues Mol Biol 2001 3 4 91 97 11719972

[B37] Comeron JM Aguade M An evaluation of measures of synonymous codon usage bias J Mol Evol 1998 47 3 268 274 10.1007/PL00006384 9732453

[B38] Bofkin L Goldman N Variation in evolutionary processes at different codon positions Mol Biol Evol 2007 24 2 513 521 17119011 10.1093/molbev/msl178

[B39] Karlin S Global dinucleotide signatures and analysis of genomic heterogeneity Curr Opin Microbiol 1998 1 5 598 610 10.1016/S1369-5274(98)80095-7 10066522

[B40] Davis JJ Olsen GJ Characterizing the native codon usages of a genome: an axis projection approach Mol Biol Evol 2011 28 1 211 221 10.1093/molbev/msq185 20679093 PMC3002238

[B41] Mrazek J Karlin S Strand compositional asymmetry in bacterial and large viral genomes Proc Natl Acad Sci USA 1998 95 7 3720 3725 10.1073/pnas.95.7.3720 9520433 PMC19903

[B42] Oliver JL Bernaola-Galvan P Carpena P Roman-Roldan R Isochore chromosome maps of eukaryotic genomes Gene 2001 276 1-2 47 56 10.1016/S0378-1119(01)00641-2 11591471

[B43] Zhang Z Yu J Modeling compositional dynamics based on GC and purine contents of protein-coding sequences Biol Direct 2010 5 1 63 10.1186/1745-6150-5-63 21059261 PMC2989939

[B44] Biro JC Benyo B Sansom C Szlavecz A Fordos G Micsik T Benyo Z A common periodic table of codons and amino acids Biochem Biophys Res Commun 2003 306 2 408 415 10.1016/S0006-291X(03)00974-4 12804578

[B45] Zhang Z Yu J On the organizational dynamics of the genetic code Genomics Proteomics Bioinformatics 2011 9 1-2 21 29 10.1016/S1672-0229(11)60004-1 21641559 PMC5054158

[B46] Baeza-Yates R Ribeiro-Neto B Modern information retrieval 1999 New York: ACM Press

[B47] Efron B Tibshirani R An introduction to the bootstrap 1993 New York: Chapman & Hall

[B48] Hu J Zhao X Zhang Z Yu J Compositional dynamics of guanine and cytosine content in prokaryotic genomes Res Microbiol 2007 158 4 363 370 10.1016/j.resmic.2007.02.007 17449227

[B49] Duret L Mouchiroud D Expression pattern and, surprisingly, gene length shape codon usage in Caenorhabditis, Drosophila, and Arabidopsis Proc Natl Acad Sci USA 1999 96 8 4482 4487 10.1073/pnas.96.8.4482 10200288 PMC16358

[B50] Castillo-Davis CI Hartl DL Genome evolution and developmental constraint in Caenorhabditis elegans Mol Biol Evol 2002 19 5 728 735 10.1093/oxfordjournals.molbev.a004131 11961106

[B51] Wright SI Yau CB Looseley M Meyers BC Effects of gene expression on molecular evolution in Arabidopsis thaliana and Arabidopsis lyrata Mol Biol Evol 2004 21 9 1719 1726 10.1093/molbev/msh191 15201397

[B52] Bernstein JA Khodursky AB Lin PH Lin-Chao S Cohen SN Global analysis of mRNA decay and abundance in Escherichia coli at single-gene resolution using two-color fluorescent DNA microarrays Proc Natl Acad Sci USA 2002 99 15 9697 9702 10.1073/pnas.112318199 12119387 PMC124983

[B53] Holstege FC Jennings EG Wyrick JJ Lee TI Hengartner CJ Green MR Golub TR Lander ES Young RA Dissecting the regulatory circuitry of a eukaryotic genome Cell 1998 95 5 717 728 10.1016/S0092-8674(00)81641-4 9845373

[B54] Zhang Y Sturgill D Parisi M Kumar S Oliver B Constraint and turnover in sex-biased gene expression in the genus Drosophila Nature 2007 450 7167 233 237 10.1038/nature06323 17994089 PMC2386141

[B55] Roy PJ Stuart JM Lund J Kim SK Chromosomal clustering of muscle-expressed genes in Caenorhabditis elegans Nature 2002 418 6901 975 979 12214599 10.1038/nature01012

[B56] Wuest SE Vijverberg K Schmidt A Weiss M Gheyselinck J Lohr M Wellmer F Rahnenfuhrer J von Mering C Grossniklaus U Arabidopsis female gametophyte gene expression map reveals similarities between plant and animal gametes Curr Biol 2010 20 6 506 512 10.1016/j.cub.2010.01.051 20226671

[B57] Wong GK Wang J Tao L Tan J Zhang J Passey DA Yu J Compositional gradients in Gramineae genes Genome Res 2002 12 6 851 856 10.1101/gr.189102 12045139 PMC1383739

[B58] Zhang Z Yu J On the organizational dynamics of the genetic code Genomics Proteomics Bioinformatics 2010 in press 10.1016/S1672-0229(11)60004-1 PMC5054158 21641559

[B59] Qu H Wu H Zhang T Zhang Z Hu S Yu J Nucleotide compositional asymmetry between the leading and lagging strands of eubacterial genomes Res Microbiol 2010 161 10 838 846 10.1016/j.resmic.2010.09.015 20868744

[B60] Yanofsky C Platt T Crawford IP Nichols BP Christie GE Horowitz H VanCleemput M Wu AM The complete nucleotide sequence of the tryptophan operon of Escherichia coli Nucleic Acids Res 1981 9 24 6647 6668 10.1093/nar/9.24.6647 7038627 PMC327632

[B61] Karlin S Mrazek J Campbell AM Codon usages in different gene classes of the Escherichia coli genome Mol Microbiol 1998 29 6 1341 1355 10.1046/j.1365-2958.1998.01008.x 9781873

[B62] Eistetter AJ Butler PD Traut RR Fanning TG Characterization of Escherichia coli 50S ribosomal protein L31 FEMS Microbiol Lett 1999 180 2 345 349 10.1111/j.1574-6968.1999.tb08816.x 10556732

[B63] Muralikrishna P Cooperman BS Ribosomal components neighboring the 2475 loop in Escherichia coli 50S subunits Biochemistry 1995 34 1 115 121 10.1021/bi00001a014 7529559

[B64] Ikegami A Nishiyama K Matsuyama S Tokuda H Disruption of rpmJ encoding ribosomal protein L36 decreases the expression of secY upstream of the spc operon and inhibits protein translocation in Escherichia coli Biosci Biotechnol Biochem 2005 69 8 1595 1602 10.1271/bbb.69.1595 16116291

