# Supplementary material for: Codon Deviation Coefficient: a novel measure for estimating codon usage bias and its statistical significance
Source: BMC Bioinformatics. 2012 Mar 22;13:43. doi: 10.1186/1471-2105-13-43 (PMC3368730; doi:10.1186/1471-2105-13-43)
Supplement: Additional file 1 — Empirical expression data analysis. Correlations between codon usage bias and gene expression level in different expression data sets. [file 1471-2105-13-43-S1.DOC]

# Additional File 1

[Expression data collection 1](#__RefHeading___Toc301591430)

[Escherichia coli (LB medium) 3](#__RefHeading___Toc301591431)

[Escherichia coli (M9 medium) 4](#__RefHeading___Toc301591432)

[Saccharomyces cerevisiae 5](#__RefHeading___Toc301591433)

[Drosophila melanogaster 6](#__RefHeading___Toc301591434)

[Caenorhabditis elegans 7](#__RefHeading___Toc301591435)

[Arabidopsis thaliana 8](#__RefHeading___Toc301591436)

[Correlation between CDC and Scaled N_c_′ 9](#__RefHeading___Toc301591437)

##### Expression data collection

Expression data sets from five different species were collected in this study.

(1) *Escherichia coli* from Bernstein et al [1]: 1762 genes in LB medium and 2766 genes in M9 medium (Supporting Table 6; http://www.pnas.org/content/99/15/9697/suppl/DC1);

(2) *Saccharomyces cerevisiae* from Holstege et al [2] : 5142 genes (http://web.wi.mit.edu/young/expression);

(3) *Drosophila melanogaster* from Zhang et al [3]: 1651 genes (Supplementary Table S4; http://www.nature.com/nature/journal/v450/n7167/suppinfo/nature06323.html);

(4) *Caenorhabditis elegans* from Roy et al [4]: 12184 genes, with averaged expression levels across six repeats and exclusive of genes containing “NaN” (Supplementary Table S1; http://www.nature.com/nature/journal/v418/n6901/extref/nature01012-s1.htm);

(5) *Arabidopsis thaliana* from Wuest et al [5]: 1332 genes, with averaged expression levels across three cell types (each cell type has three replicates) (Supplementary Table S3 Sheet 1; http://www.sciencedirect.com/science/article/pii/S0960982210001405);

Codon usage biases estimated by CDC, Scaled *N_c_′*, Scaled *N_c_* and CAI, were plotted against gene expression levels in Figure S1-S12.

##### Escherichia coli (LB medium)


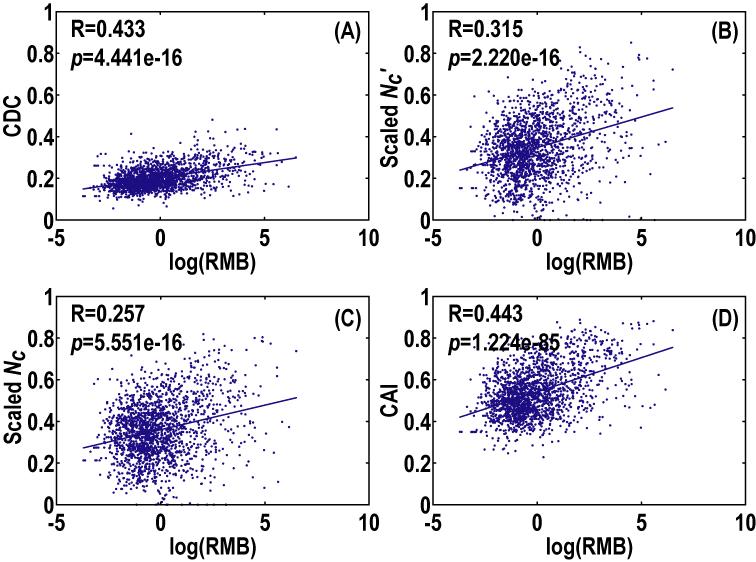


1. Correlations between relative molecular abundance (RMB) values of 1762 *E. coli* genes in LB medium and codon usage biases estimated by CDC (A), Scaled *N_c_′* (B), Scaled *N_c_* (C), and CAI (D). RMB values were retrieved from Bernstein et al [1] and plotted in logarithmic scale.


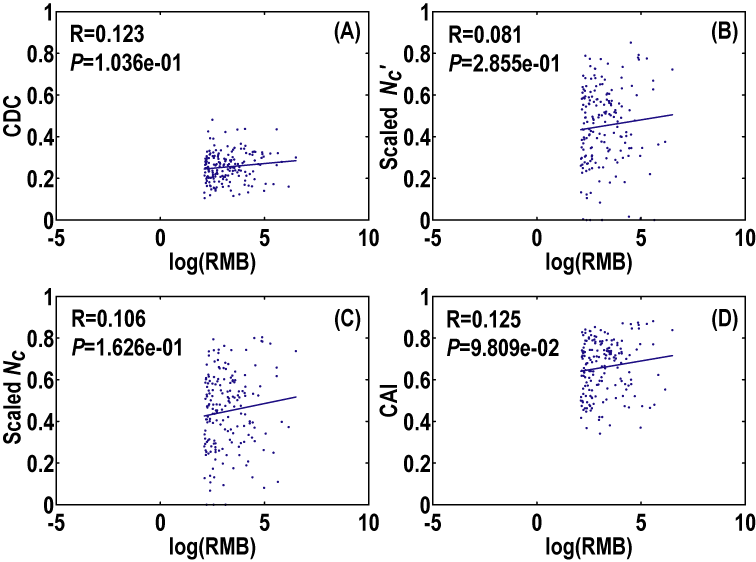


1. Correlations between top 10% relative molecular abundance (RMB) values of 1762 *E. coli* genes in LB medium and codon usage biases estimated by CDC (A), Scaled *N_c_′* (B), Scaled *N_c_* (C), and CAI (D). RMB values were retrieved from Bernstein et al [1] and plotted in logarithmic scale.

##### Escherichia coli (M9 medium)


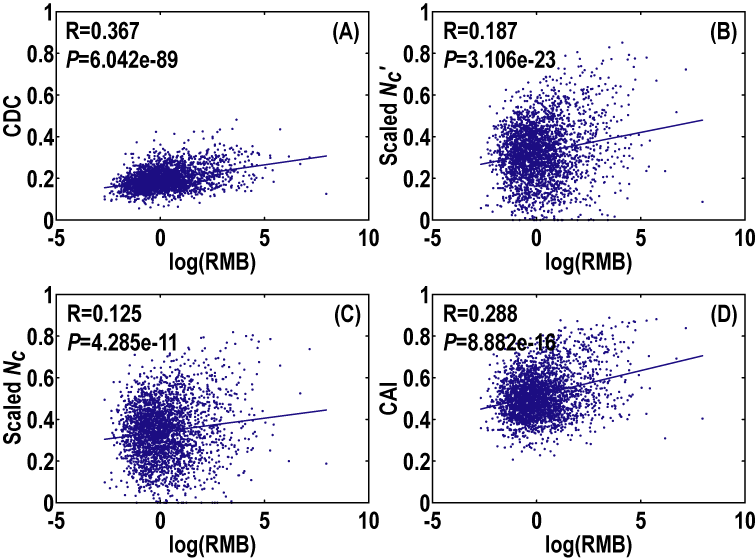


1. Correlations between relative molecular abundance (RMB) values of 2766 *E. coli* genes in M9 medium and codon usage biases estimated by CDC (A), Scaled *N_c_′* (B), Scaled *N_c_* (C), and CAI (D). RMB values were retrieved from Bernstein et al [1] and plotted in logarithmic scale.


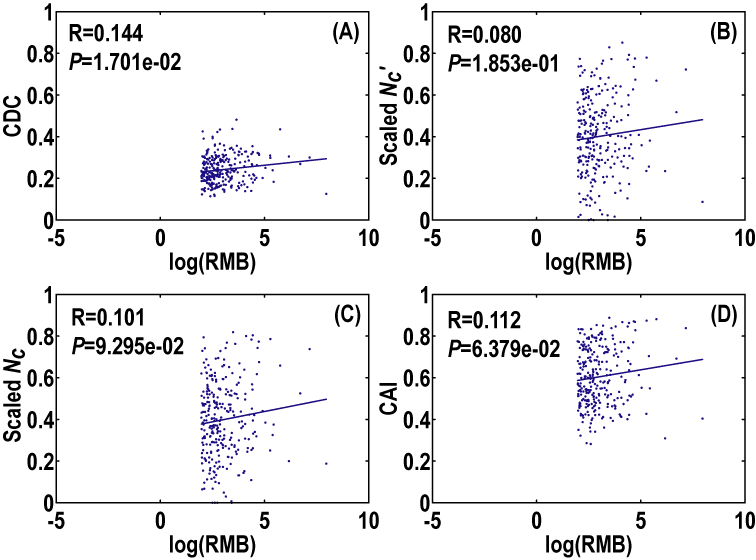


1. Correlations between top 10% relative molecular abundance (RMB) values of 2766 *E. coli* genes in M9 medium and codon usage biases estimated by CDC (A), Scaled *N_c_′* (B), Scaled *N_c_* (C), and CAI (D). RMB values were retrieved from Bernstein et al [1] and plotted in logarithmic scale.

##### Saccharomyces cerevisiae


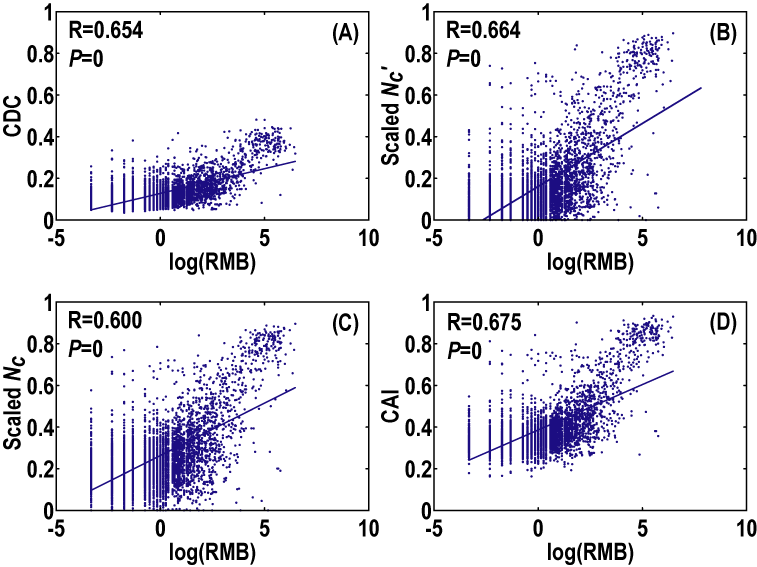


1. Correlations between relative molecular abundance (RMB) values of 5142 *S. cerevisiae* genes and codon usage biases estimated by CDC (A), Scaled *N_c_′* (B), Scaled *N_c_* (C), and CAI (D). RMB values were retrieved from Holstege et al. [2] and plotted in logarithmic scale.


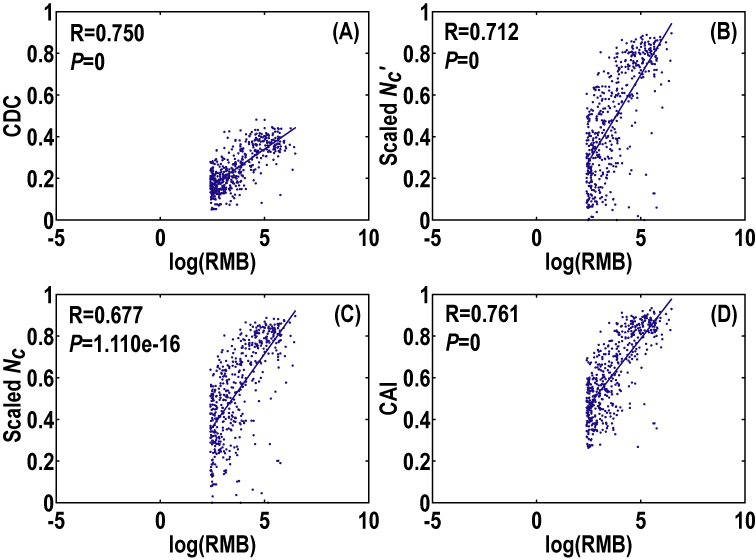


1. Correlations between top 10% relative molecular abundance (RMB) values of 5142 *S. cerevisiae* genes and codon usage biases estimated by CDC (A), Scaled *N_c_′* (B), Scaled *N_c_* (C), and CAI (D). RMB values were retrieved from Holstege et al. [2] and plotted in logarithmic scale.

##### Drosophila melanogaster


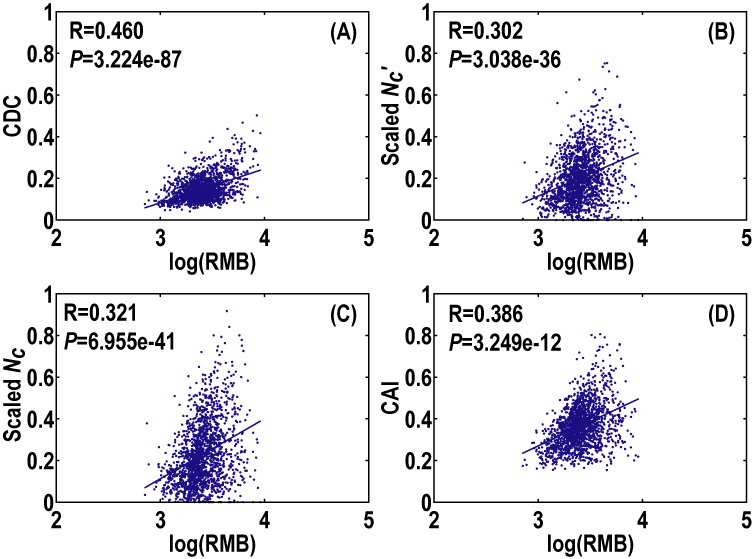


1. Correlations between relative molecular abundance (RMB) values of 1651 *D. melanogaster* genes and codon usage biases estimated by CDC (A), Scaled *N_c_′* (B), Scaled *N_c_* (C), and CAI (D). RMB values were retrieved from Zhang et al [3] and plotted in logarithmic scale.


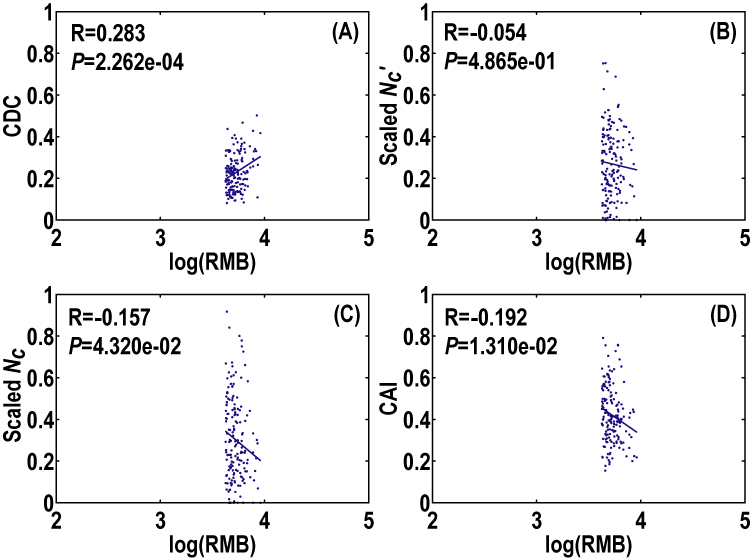


1. Correlations between top 10% relative molecular abundance (RMB) values of 1651 *D. melanogaster* genes and codon usage biases estimated by CDC (A), Scaled *N_c_′* (B), Scaled *N_c_* (C), and CAI (D). RMB values were retrieved from Zhang et al [3] and plotted in logarithmic scale.

##### Caenorhabditis elegans


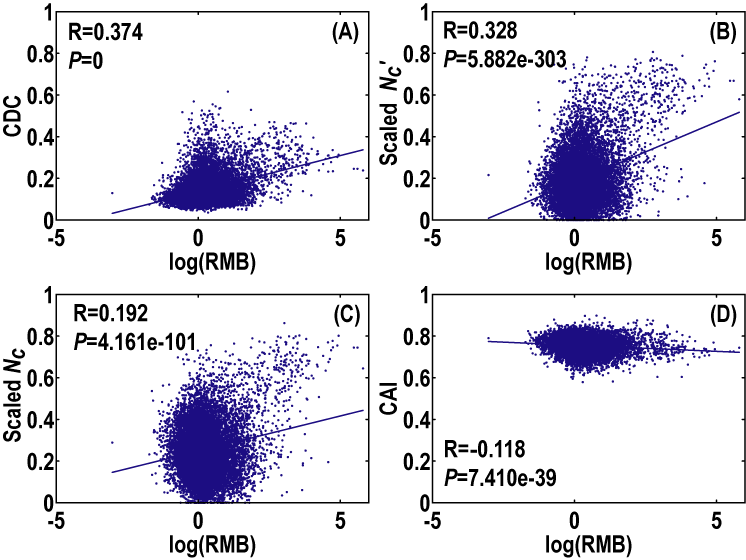


1. Correlations between relative molecular abundance (RMB) values of 12184 *C. elegans* genes and codon usage biases estimated by CDC (A), Scaled *N_c_′* (B), Scaled *N_c_* (C), and CAI (D). RMB values were retrieved from Roy et al [4] and plotted in logarithmic scale.


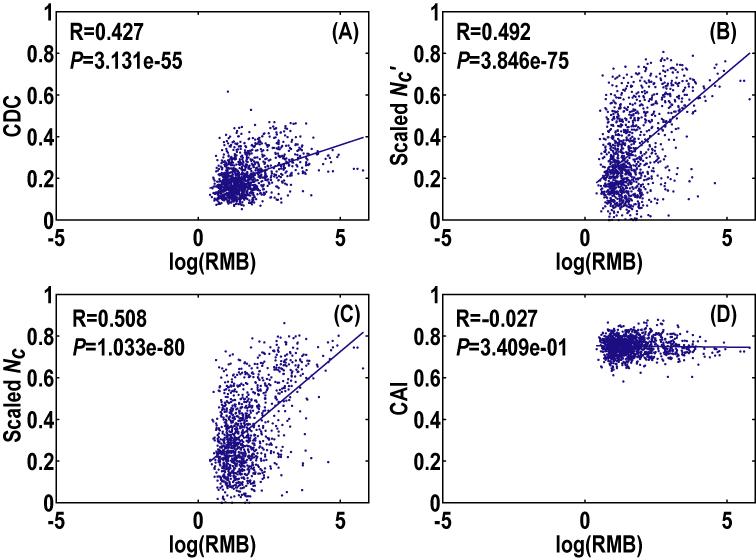


1. Correlations between top 10% relative molecular abundance (RMB) values of 12184 *C. elegans* genes and codon usage biases estimated by CDC (A), Scaled *N_c_′* (B), Scaled *N_c_* (C), and CAI (D). RMB values were retrieved from Roy et al [4] and plotted in logarithmic scale.

##### Arabidopsis thaliana


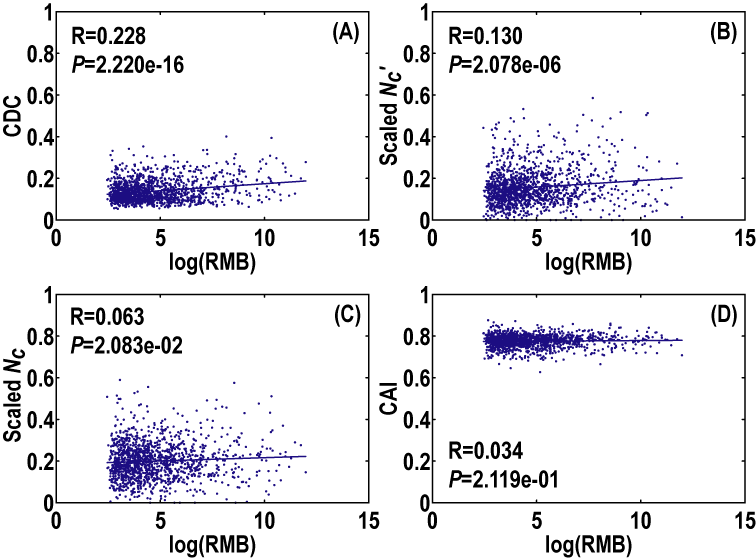


1. Correlations between relative molecular abundance (RMB) values of 1332 *A. thaliana* genes and codon usage biases estimated by CDC (A), Scaled *N_c_′* (B), Scaled *N_c_* (C), and CAI (D). RMB values were retrieved from Wuest et al [5] and plotted in logarithmic scale.


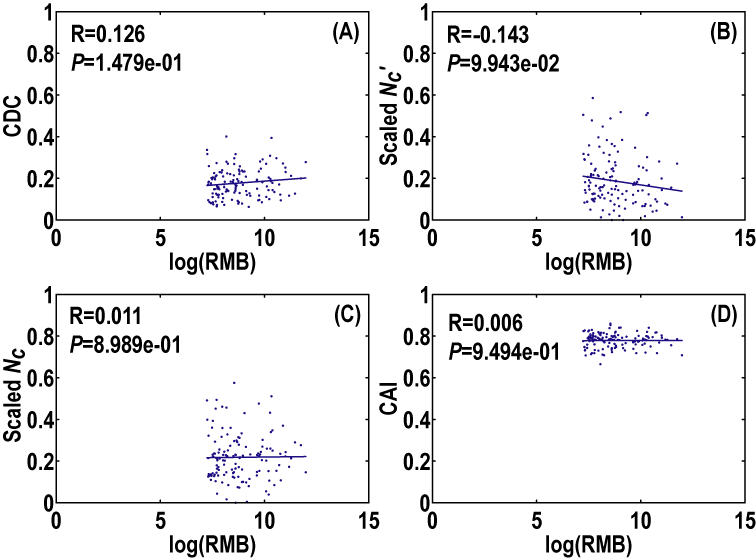


1. Correlations between top 10% relative molecular abundance (RMB) values of 1332 *A. thaliana* genes and codon usage biases estimated by CDC (A), Scaled *N_c_′* (B), Scaled *N_c_* (C), and CAI (D). RMB values were retrieved from Wuest et al [5] and plotted in logarithmic scale.

##### Correlation between CDC and Scaled N_c_′


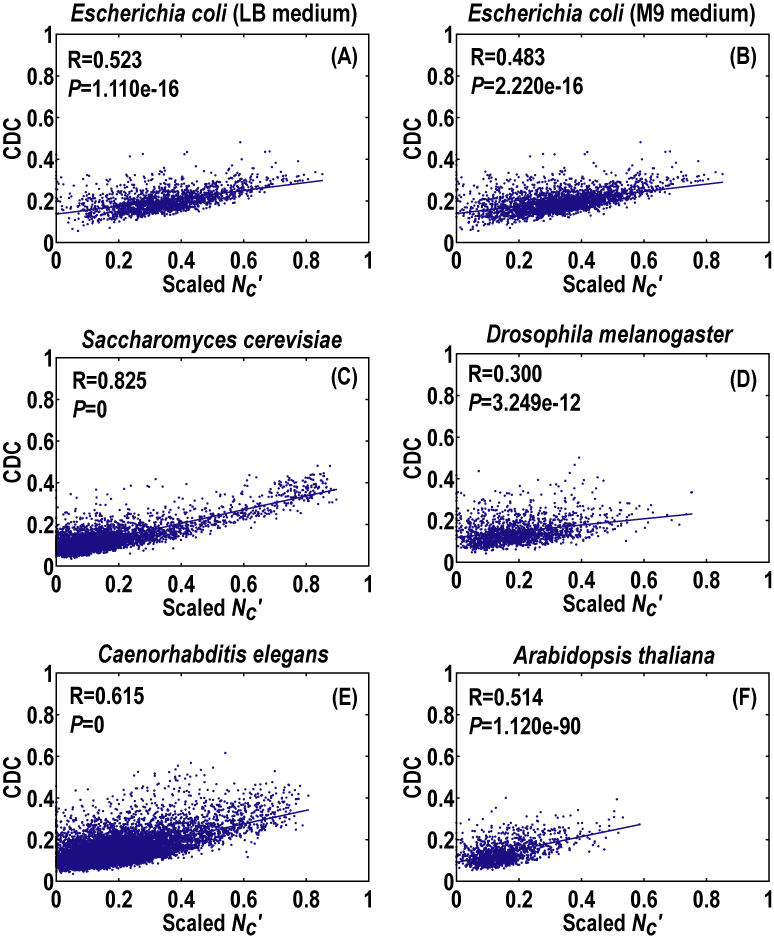


1. Correlations between CDC and Scaled *N_c_′*. (A) *E. coli*: 1762 genes in LB medium; (B) *E. coli*: 2766 genes in M9 medium; (C) *S. cerevisiae*: 5142 genes; (D) *D. melanogaster*: 1651 genes; (E) *C. elegans*: 12184 genes; and (F) *A. thaliana*: 1332 genes.

### References

1. Bernstein JA, Khodursky AB, Lin PH, Lin-Chao S, Cohen SN: **Global analysis of mRNA decay and abundance in Escherichia coli at single-gene resolution using two-color fluorescent DNA microarrays**. *Proc Natl Acad Sci U S A* 2002, **99**(15):9697-9702.

2. Holstege FC, Jennings EG, Wyrick JJ, Lee TI, Hengartner CJ, Green MR, Golub TR, Lander ES, Young RA: **Dissecting the regulatory circuitry of a eukaryotic genome**. *Cell* 1998, **95**(5):717-728.

3. Zhang Y, Sturgill D, Parisi M, Kumar S, Oliver B: **Constraint and turnover in sex-biased gene expression in the genus Drosophila**. *Nature* 2007, **450**(7167):233-237.

4. Roy PJ, Stuart JM, Lund J, Kim SK: **Chromosomal clustering of muscle-expressed genes in Caenorhabditis elegans**. *Nature* 2002, **418**(6901):975-979.

5. Wuest SE, Vijverberg K, Schmidt A, Weiss M, Gheyselinck J, Lohr M, Wellmer F, Rahnenfuhrer J, von Mering C, Grossniklaus U: **Arabidopsis female gametophyte gene expression map reveals similarities between plant and animal gametes**. *Curr Biol* 2010, **20**(6):506-512.
